# The circadian clock mediates daily bursts of cell differentiation by periodically restricting cell-differentiation commitment

**DOI:** 10.1073/pnas.2204470119

**Published:** 2022-08-08

**Authors:** Zhi-Bo Zhang, Joydeb Sinha, Zahra Bahrami-Nejad, Mary N. Teruel

**Affiliations:** ^a^Department of Biochemistry, Weill Cornell Medical College of Cornell University, New York, NY 10065;; ^b^The Ira & Gale Drukier Institute of Children’s Health, Weill Cornell Medical College of Cornell University, New York, NY 10065;; ^c^Department of Chemical and Systems Biology, Stanford University School of Medicine, Stanford, CA 94305;; ^d^Weill Center for Metabolic Health, Division of Endocrinology, Diabetes, and Metabolism, Joan and Sanford I. Weill Department of Medicine, Weill Cornell Medical College of Cornell University, New York, NY 10065;; ^e^Department of Bioengineering, Stanford University, Stanford, CA 94305

**Keywords:** circadian rhythms, adipogenesis, cell differentiation, positive feedback, cell-fate decision

## Abstract

Most mammalian cells have an intrinsic circadian clock that coordinates metabolic activity with the daily rest and wake cycle. The circadian clock is known to regulate cell differentiation, but how continuous daily oscillations of the internal clock can control a much longer, multiday differentiation process is not known. Here, we simultaneously monitor circadian clock and adipocyte-differentiation progression live in single cells. Strikingly, we find a bursting behavior in the cell population whereby individual preadipocytes commit to differentiate primarily during a 12-h window each day, corresponding to the time of rest. Daily gating occurs because cells irreversibly commit to differentiate within only a few hours, which is much faster than the rest phase and the overall multiday differentiation process. The daily bursts in differentiation commitment result from a differentiation-stimulus driven variable and slow increase in expression of PPARG, the master regulator of adipogenesis, overlaid with circadian boosts in PPARG expression driven by fast, clock-driven PPARG regulators such as CEBPA. Our finding of daily bursts in cell differentiation only during the circadian cycle phase corresponding to evening in humans is broadly relevant, given that most differentiating somatic cells are regulated by the circadian clock. Having a restricted time each day when differentiation occurs may open therapeutic strategies to use timed treatment relative to the clock to promote tissue regeneration.

## Significance

Cells rely on a circadian clock that coordinates cellular activities with the day–night cycle. Defects in circadian clock genes dysregulate cell-differentiation processes in immune, muscle, skin, and fat cells. However, how a fast, invariable daily clock can regulate a slow, multiday cell-differentiation process was not understood. Here, we show that, even though differentiation occurs over days, differentiation commitment occurs rapidly in only a few hours, much faster than the 24-hour rhythm of the circadian clock and thus allowing differentiation to be gated by the clock. Our finding of daily bursts of cell differentiation restricted to the evening phase opens up potential therapeutic strategies to control tissue regeneration by timing when during the day drugs are administered.

Virtually all cells in the human body contain an intrinsic circadian clock (cell-intrinsic clock), operated by a set of core clock proteins that engage in coupled positive and negative transcriptional and translational feedback loops to generate rhythmic expression of 10 to 15% of the transcriptome (1). When components that drive the cell-intrinsic clock are genetically perturbed, cell differentiation of fat cells (adipocytes) (2, 3), T cells (4), myoblasts (5), and embryonic stem cells (6) are defective, suggesting that the circadian clock regulates differentiation. However, it is not clear how a daily clock that oscillates perpetually can control a much slower process such as cell differentiation, which typically takes several days or even weeks.

One possibility is that cells count the number of circadian cycles to delay differentiation for a certain time period after the differentiation stimulus is added. Another possibility is that there may be a time window during each circadian cycle in which cells can commit to differentiate; the differentiation process is prolonged if a cell misses to commit in this time window and needs to wait for a subsequent permissive window. To distinguish between these and other possible mechanisms, we used adipogenesis as a model system since it is currently the only differentiation system for which validated tools are available to measure in live cells the time when cells irreversibly differentiate. Our strategy builds on a previously developed method to track cell-differentiation progression by monitoring the endogenous expression of PPARG, the master regulator of adipogenesis, over several days (7, 8). The time when a preadipocyte irreversibly differentiates, called the differentiation-commitment point, can be measured as the time when the PPARG level in the cell increases to a critical threshold at which positive-feedback loops engage to lock the PPARG level at a perpetually high level (7, 8). Here, we sought to understand how the circadian clock controls differentiation by using this cell model to measure the circadian clock and differentiation commitment live in the same cell.

Strikingly, rather than finding evidence for counting of circadian cycles, we found that preadipocytes commit to differentiate in repeated daily bursts that occur exclusively during the phase of the circadian cycle that matches the resting period in humans (9). Mechanistically, we show that circadian expression of CEBPA, a positive-feedback regulator of PPARG, controls a periodic increase in PPARG, which then triggers differentiation only if PPARG reaches the threshold during the resting phase of the circadian cycle. Even though the overall differentiation process takes many days to complete, irreversible commitment to differentiate occurs rapidly, as demonstrated by a switch from low-to-permanently high PPARG levels within approximately 4 hours when a preadipocyte commits to differentiate. It is this fast commitment step that is gated by the 12-h pattern of the circadian clock, explaining how circadian rhythms can control a differentiation process that takes many days. Our study argues that the cell-intrinsic circadian clock controls cell differentiation by restricting it to a short phase window each day, providing a mechanism for how dysregulated circadian rhythms may broaden this daily phase window to increase differentiation and fat mass.

## Results

### Development of a System to Simultaneously Monitor the Cell-Intrinsic Clock and Cell-Differentiation Progression in Single Cells.

**Fig. 1.**
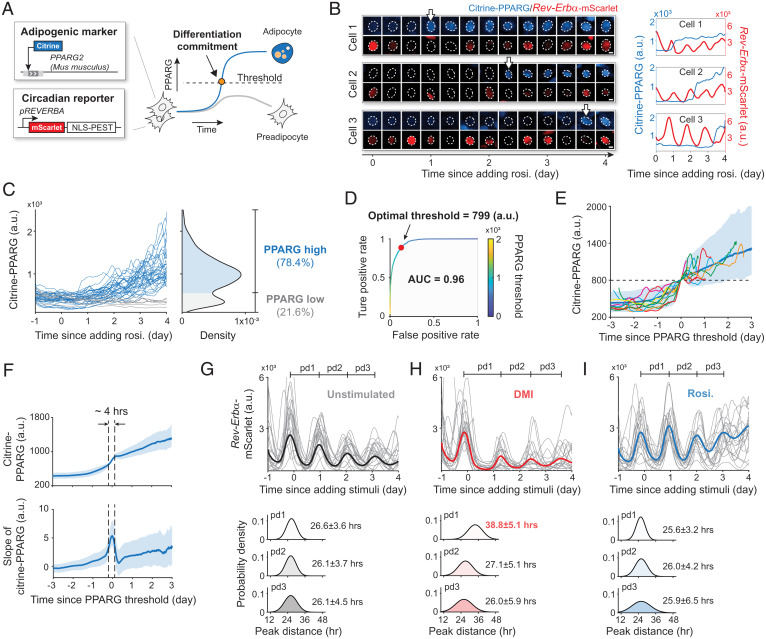
Simultaneous monitoring of the cell-intrinsic circadian clock and adipogenesis in single cells. (*A*) Schematic of the cell-model design. (*B*) The citrine–PPARG/*Rev-Erb*α–mScarlet dual-reporter cells were stimulated with 100 nM rosiglitazone (rosi.). The dotted outlines mark the nuclei. The arrows indicate the time when cells switch to the high PPARG state. (Scale bars, 5 μm.) (*C*) Single-cell time courses of citrine–PPARG were divided into two categories based on the citrine–PPARG intensity at day 4. Representative of three biological replicates. (*D*) ROC curve analysis was used to determine an optimal threshold in the PPARG level, which can predict the fate of most individual cells correctly. AUC represents the two-dimensional area underneath the entire ROC curve. (*E* and *F*) Citrine–PPARG time courses from *C* were aligned to the time when the cell reached the optimal threshold. (*E*) Plot of 10 representative aligned time courses (light lines), as well as the median (solid line) and the 5th to 95th percentiles (shaded area) from *n* = ∼13,000 differentiated cells. (*F*) Plot of median and the 25th to 75th percentiles. (*G*–*I*) Each plot shows 20 representative time courses and the median from *n* = ∼5,000 cells. The peak-to-peak distance (pd) is presented as mean ± SD. Representative of three biological replicates. A.u., arbitrary units.

Adipogenesis is a multiday process during which preadipocytes irreversibly differentiate into adipocytes, primarily through the expression of PPARG, the master regulator of fat-cell differentiation (Fig. 1*A*). Our previous studies showed that the time when a preadipocyte irreversibly commits to become an adipocyte, also known as the adipogenesis commitment point, can be precisely marked by the time when the abundance of PPARG protein reaches a threshold level (7, 8). To understand how the cell-intrinsic clock regulates the timing of adipogenesis, we used a modified version of a previously described circadian reporter (10), which comprises coding and promoter sequences of *Rev-Erb*α conjugated to mScarlet (RFP) protein. We introduced this *Rev-Erb*α circadian clock reporter into an OP9 preadipocyte cell line, in which endogenous PPARG had been tagged with citrine (YFP) using CRISPR genome editing (7). Fig. 1*B* shows example time courses of citrine–PPARG/*Rev-Erb*α–mScarlet dual reporter cells undergoing adipogenesis.

Adipogenesis is invariably a bistable process: Preadipocytes induced to differentiate end up in either a high- or low-PPARG state (7, 11), corresponding to being either differentiated or undifferentiated (Fig. 1 *A* and *C*). Upon addition of a differentiation stimulus, PPARG levels start to increase gradually in preadipocytes (7). However, preadipocytes only irreversibly commit to differentiate when PPARG levels increase to a threshold level, at which multiple positive feedbacks to PPARG engage so strongly that PPARG levels stay high, even when the differentiation stimulus is removed (7). The time when a cell reaches the threshold and irreversibly commits to differentiate can be seen by a step increase from low-to-high PPARG level (marked with white arrows in Fig. 1*B*).

To precisely calculate when a cell has reached the threshold, we used receiver operating characteristic (ROC) analysis (Fig. 1*D* and *Materials and Methods*) (12). In this analysis, different threshold levels are surveyed to find the one that maximizes the difference between true- and false-positive rates for predicting cell-fate choice (12). For the typical experiment shown in Fig. 1*D*, the area under the curve (AUC) value of 0.96 is close to the maximal value of 1, demonstrating that the optimal citrine–PPARG threshold derived from ROC analysis can be used to measure with high accuracy the precise time when cells commit to differentiate. When the citrine–PPARG traces from Fig. 1*C* are computationally aligned to the time when each cell reached the PPARG threshold, a bimodal switch from low (undifferentiated) to high (differentiated) PPARG level can be observed (Fig. 1*E*). Once this bimodal switch occurs, the cell is irreversibly committed to the differentiated state; PPARG levels remain high and never drop back down, even if the differentiation stimulus is removed (8) (Fig. 1*E*). As shown in Fig. 1 *E* and *F*, PPARG increases gradually in preadipocytes induced to differentiate, often over several days before the fast commitment step occurs. Furthermore, after the step increase in PPARG level occurs, PPARG levels often continue to increase gradually for a few more days before cells are fully differentiated. We confirmed that the step increase from low to high PPARG levels, representing differentiation commitment, occurs rapidly, within only a few hours (Fig. 1*F*).

Using the *Rev-Erb*α–mScarlet reporter to analyze the circadian clock dynamics, we found that the circadian period was ∼26 h in unstimulated control cells, (Fig. 1*G*). Adipogenic stimuli often contain added glucocorticoids that promote differentiation, but also reset the circadian clock in peripheral cells and tissues (13). Indeed, we confirmed that applying the commonly used DMI (dexamethasone, 3-isobutyl-1-methylxanthine [IBMX], and insulin) stimulus that contains a synthetic glucocorticoid, dexamethasone, perturbs the circadian clock by delaying the first peak of the *Rev-Erb*α reporter by ∼12 h, after which the peak-to-peak distances return to ∼26 h (Fig. 1*H*). Thus, to prevent resetting the clock during our analysis of differentiation, we instead used the PPARG agonist rosiglitazone to induce adipogenesis (14, 15) (*SI Appendix*, Fig. S1), which kept the circadian clock period at ∼26 h over the several-day time course of adipogenesis (Fig. 1*I*).

### Differentiation Commitment Is Almost Exclusively Triggered during the Rising Phase of the *Rev-Erb*α Reporter.

**Fig. 2.**
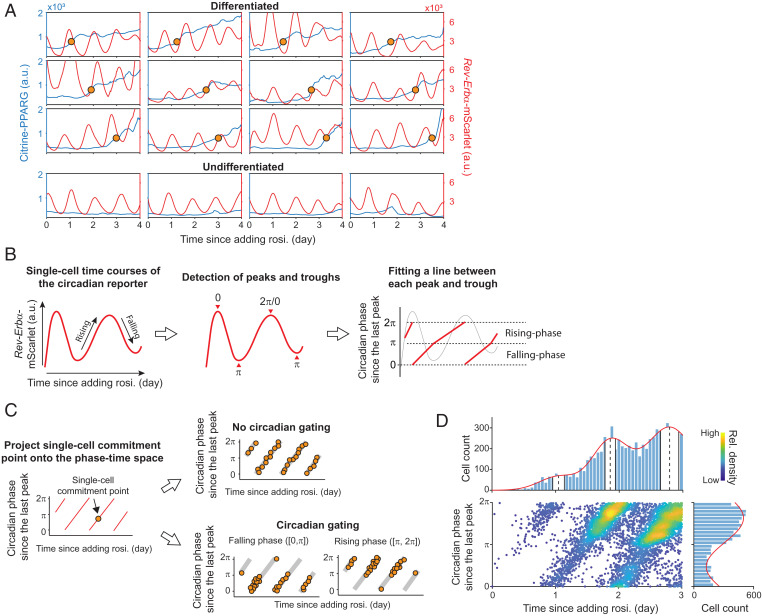
The timing of differentiation commitment follows a circadian pattern. (*A*) Examples of single-cell time courses of citrine–PPARG (blue) and circadian reporter (red) in response to 100 nM rosiglitazone (rosi.). For differentiated cells, the yellow circle marks the time when the cell reaches the PPARG threshold and commits to the differentiated state. (*B*) Scheme showing procedure to convert the single-cell time courses of the *Rev-Erb*α reporter into the circadian phase. (*C*) Scheme showing how to determine if and how the cell-intrinsic clock regulates the timing of differentiation commitment. (*D*) The scatterplot represents the distribution of commitment points in the phase–time space. *n* = ∼13,000. (*Upper*) The distribution of commitment points over time was fitted by a Gaussian mixture model. Vertical dashed lines and white bands indicate µ (mean) and σ (SD) for the first three components, respectively. (*Lower*) Distribution of commitment-point phases was fitted by a one-term Fourier model. Representative of three biological replicates. A.u., arbitrary units; rel., relative.

To determine when a cell commits to differentiate relative to the cell-intrinsic clock, we analyzed expression of citrine–PPARG and the *Rev-Erb*α reporter simultaneously in the same cells during adipogenesis. A visual inspection of hundreds of single-cell time courses (see representative examples in Fig. 2*A*) showed great variability in the number of circadian cycles that occur before cells commit to differentiate, ruling out the initial hypothesis that cells delay adipogenesis by counting a fixed number of oscillations. We therefore turned to the second possibility introduced above that the cell-intrinsic circadian clock may trigger commitment at a particular circadian clock phase. To determine the phase when a cell commits to differentiate, we first measured the time when each individual cell reached the PPARG threshold for irreversible commitment. We then used a customized MATLAB script to detect the peaks and troughs in the *Rev-Erb*α reporter oscillations by defining each peak as phase 0 or 2π and each trough as phase π (Fig. 2 *B*, *Center*). Using a linear fit from peaks to troughs, we then converted the time of differentiation commitment into a circadian phase relative to the cell’s last peak of the *Rev-Erb*α reporter (Fig. 2 *B*, *Right* and *SI Appendix*, Fig. S2*A*).

The scheme in Fig. 2*C* depicts how this analysis can be used to calculate a phase-corrected time of commitment. Fig. 2 *C*, *Left* shows the projection of one single-cell commitment point onto the phase–time space. By plotting the distribution of commitment points of thousands of single cells within this phase–time plot, we can determine if and when the cell-intrinsic circadian clock gates differentiation commitment. For example, if a particular time in the circadian oscillation indeed gates when cells commit to differentiate, there should be recurring bursts in the distribution in the phase–time plot during sequential circadian oscillations; otherwise, if cells commit to differentiate independently of the phase of the circadian clock, the commitment points should be evenly spaced over the circadian oscillation (Fig. 2 *C*, *Right*).

We quantitatively tested whether preadipocytes exhibit circadian gating by projecting ∼13,000 commitment points onto the phase–time plot (Fig. 2*D* and *SI Appendix*, Fig. S2*B*). Strikingly, we found that commitment was almost exclusively triggered between the π to 2π half of the circadian cycle, resulting in bursts of differentiation-commitment events that were spread over sequential circadian oscillations and provided strong evidence for circadian gating of differentiation. Fig. 2 *D*, *Upper* shows that the distribution of the commitment time peaks every day. However, only by also plotting the phase could we also learn that cells preferentially commit to differentiate every day during the rising phase of the *Rev-Erb*α reporter.

### Manipulations of Circadian Rhythms Demonstrate the Cell-Intrinsic Clock Gates the Timing of Differentiation Commitment.

**Fig. 3.**
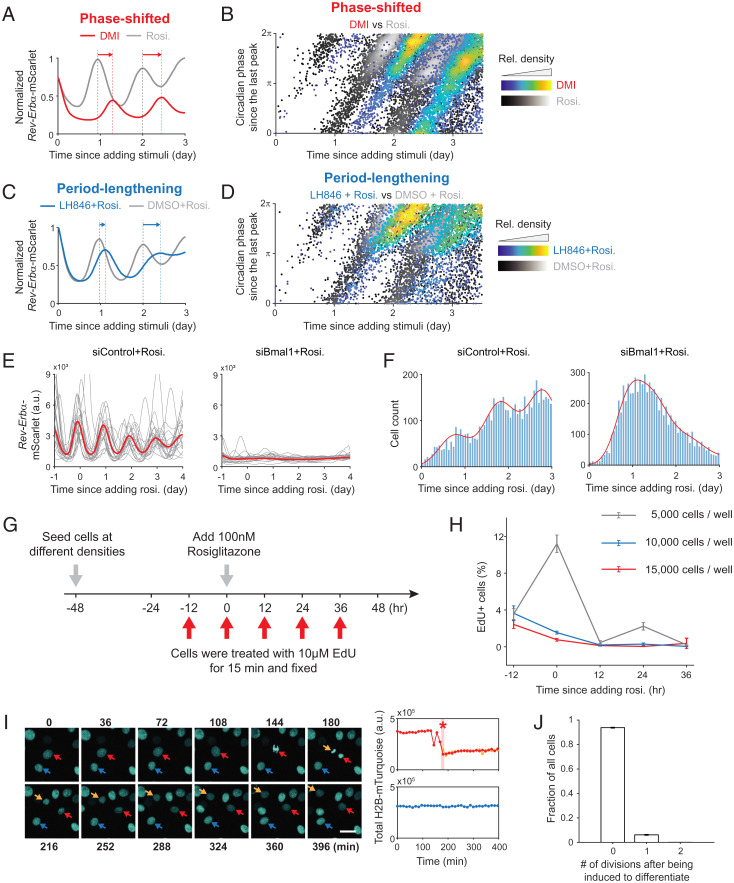
Validations of cell-intrinsic clock gating the timing of differentiation commitment. (*A*) The citrine–PPARG/*Rev-Erb*α–mScarlet dual-reporter cells were induced to differentiate by addition of a DMI mixture or 100 nM rosiglitazone (rosi). The median of *n* = ∼5,000 *Rev-Erb*α reporter time courses is normalized to the maximum value. Representative of three biological replicates. (*B*) Comparison of the commitment-point patterns in the phase–time space shows that differentiation-commitment time is pushed back by the delayed phase of the circadian clock, but the time when cells commit to differentiate remains between the π to 2π phase of the circadian reporter (also see *SI Appendix*, Fig. S3*B*). (*C*) The citrine–PPARG/*Rev-Erb*α–mScarlet dual-reporter cells were induced to differentiate by 100 nM rosiglitazone along with 4 µM LH846 or DMSO (control). The small-molecule LH846 caused the periods of the circadian clock to be lengthened. The median of *n* = ∼6,000 *Rev-Erb*α reporter time courses is normalized to the maximum value. Representative of three biological replicates. (*D*) The period lengthening caused by the small-molecule LH846 resulted in delayed differentiation commitment, but the phases of the commitment points are still enriched in the range from π to 2π (also see *SI Appendix*, Fig. S3*D*). (*E* and *F*) The citrine–PPARG/*Rev-Erb*α–mScarlet dual-reporter cells transfected with BMAL1 or nontargeting siRNA were stimulated with 100 nM rosiglitazone. (*E*) Each plot shows 20 representative time courses and the median from *n* = ∼11,000 cells. (*F*) The distribution of commitment points over time was fitted by a Gaussian mixture model. Representative of three biological replicates. (*G*) The citrine–PPARG/*Rev-Erb*α–mScarlet dual-reporter cells seeded at different densities were incubated with EdU at indicated times prior to fixing. (*H*) Each bar represents ∼6,000 cells from three separate wells (mean ± SD of three replicate wells). (*I*) The total H2B-mTurquoise fluorescence was quantified in the tracked nuclei. The arrows indicate three representative cells, with the blue one being for a nondivided cell and the red and orange ones being for the divided cells. The asterisk indicates the first frame postmitosis. (Scale bar, 15 μm.) (*J*) A total of 15,000 cells per well were plated 1 d prior to the typical 5-d live-cell-imaging experiment. The number of divisions for each individual cell was counted based on the time course of total H2B-mTurquoise (also see *SI Appendix*, Fig. S4). Each bar represents ∼6,000 cells from three biological replicates (mean ± SD of three biological replicates). A.u., arbitrary units; rel., relative.

Having established that differentiation commitment occurs in bursts that correlate with sequential rising phases of the circadian reporter (Fig. 2*D*), we next pharmacologically manipulated the circadian oscillation waveforms during differentiation to test whether the rhythms of the circadian clock were indeed controlling the observed gating of differentiation. We first used the commonly used adipogenic mixture DMI, which contains dexamethasone, a potent glucocorticoid that delays the first clock oscillation by ∼12 h (Figs. 1*G* and 3*A* and *SI Appendix*, Fig. S3*A*). As shown in Fig. 3*B*, the bursts of differentiation commitment when DMI was added were also shifted by 12 h. However, even though the differentiation bursts were shifted in time, the phase when cells commit to differentiate remained between π and 2π (Fig. 3*B* and *SI Appendix*, Fig. S3*B*), supporting that the rising phase of the *Rev-Erb*α reporter controls differentiation commitment.

To further validate the role of circadian phase in controlling differentiation commitment, we treated differentiating cells with LH846, a small molecule that lengthens circadian cycles by inhibiting the endogenous degradation of PER proteins (16). Consistent with circadian gating, LH846 gradually delayed the peaks of the *Rev-Erb*α circadian reporter (Fig. 3*C* and *SI Appendix*, Fig. S3*C*), but differentiation commitment still occurred tightly between π and 2π of the circadian oscillations (Fig. 3*D* and *SI Appendix*, Fig. S3*D*).

As another control to show that the phase of the clock gates differentiation commitment, we used short interfering RNA (siRNA) to deplete expression of Bmal1, a key component of the clock, in the dual-tagged cells and then induced differentiation by adding rosiglitazone. Knocking down Bmal1 abolished circadian rhythms (Fig. 3*E*). Since there are no clock phases, cells committed to differentiate in one peak instead of in multiple daily bursts (Fig. 3*F*).

Since the cell cycle has been shown to influence the circadian clock (17) and adipogenesis (8), we purposely plated the cells at very high density (15,000 cells per well) to minimize cell proliferation during the differentiation process. We then used two different methods to verify that differences in cell-cycle phases are likely not responsible for the gating. First, we carried out 5-ethynyl-2′-deoxyuridine (EdU) measurements, which showed that less than 1% of cells entered S phase at multiple time points after being induced to differentiate (Fig. 3*H*). Second, we used the nuclear marker H2B-mTurquoise in live-cell imaging experiments and showed that less than 5% of cells underwent cell division during the 4-d live-cell differentiation experiment (18) (Fig. 3 *I* and *J*). Furthermore, the few cells that did divide did so only once and only very early in the differentiation process, days before differentiation commitment typically occurs (8) (*SI Appendix*, Fig. S4 and Movie S1).

Taken together, we conclude that differentiation commitment occurs in sequential daily bursts gated by the circadian clock. Since the rising phase of the *Rev-Erb*α reporter corresponds to the sleep/inactive cycle for both diurnal and nocturnal animals (9, 19–21), our results suggest that preadipocytes commit to differentiate primarily during the evening for humans and during the day for mice.

### Circadian Regulation of CEBPA Triggers Bursts of Commitment during Adipogenesis.

**Fig. 4.**
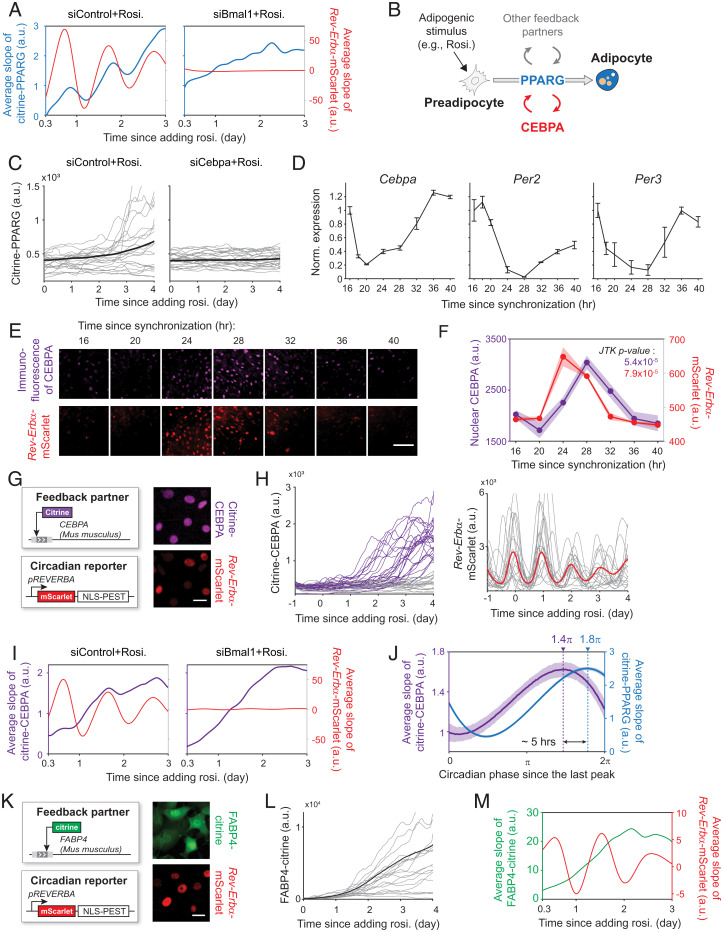
Both PPARG and its feedback partner CEBPA are expressed in a circadian pattern during adipogenesis. (*A*) The average slope of PPARG time courses from *n* = ∼7,000 cells shows that the PPARG synthesis rate follows a circadian pattern. Representative of three biological replicates. (*B*) Schematic of canonical regulatory circuits controlling the expression of PPARG during adipogenesis. (*C*) The citrine–PPARG/Rev-Erbα–mScarlet dual-reporter cells transfected with CEBPA or nontargeting siRNA were stimulated with 100 nM rosiglitazone (rosi). Each plot shows 20 representative time courses and the median from *n* = ∼16,000 cells. (*D–F*) The circadian clock was synchronized by a 1-h dexamethasone pulse. After 16 h, cell harvest for RNA extraction or cell fixation for immunofluorescent staining was performed at the indicated times. (*D*) Bar plot represents mean ± SD of three technical repeats. (*E*) (Scale bar, 100 μm.) (*F*) Shaded region represents mean ± SD of three technical repeats with *n* = ∼10,000 cells each. (*G*) System to simultaneously monitor the dynamics of CEBPA and circadian rhythm. (Scale bar, 10 μm.) (*H*) The citrine–CEBPA/*Rev-Erb*α–mScarlet dual-readout cells were induced to differentiate by using 100 nM rosiglitazone. (*H*, *Left*) Single-cell time courses of citrine–CEBPA can be divided into two categories (purple and gray) based on the nuclear citrine–CEBPA intensity at day 4. (*H*, *Right*) Plot shows 20 representative time courses and the median from *n* = ∼5,000 cells. (*I*) The average slope of CEBPA time courses of *n* = ∼5,000 cells shows that CEBPA synthesis rate also follows a circadian pattern. Representative of three biological replicates. (*J*) Single-cell time courses were computationally aligned and scaled to circadian phase from 0 to 2π for each period. Plotted are the mean and the 95% CIs generated from 1,000 bootstrap resampling at each timepoint. Dashed lines indicate the circadian phase of the maximum slope values. (*K*) The endogenous FABP4 in OP9 cells was tagged with a fluorescent protein citrine. (Scale bar, 10 μm.) (*L*) Cells were induced to differentiate by the addition of 100 nM rosiglitazone. Plot shows 20 representative time courses and the median from *n* = ∼6,000 cells. (*M*) The average slope of FABP4 time courses of *n* = ∼6,000 cells shows that FABP4 synthesis rate does not follow a circadian pattern. Representative of three biological replicates. A.u., arbitrary units; norm., normalized.

As shown in Fig. 1*E*, differentiation commitment occurs when the abundance of PPARG increases to a threshold level. Thus, in order for differentiation commitment to be gated during the π to 2π phase of the *Rev-Erb*α sensor, PPARG abundance can only be boosted to the threshold during this 12-h window in each circadian cycle. Since potential circadian oscillations in PPARG synthesis rate are masked by the gradual overall increase in PPARG abundance during adipogenesis (Fig. 1 *C* and *F*), we averaged time courses of the *Rev-Erb*α reporter and PPARG abundance, respectively, from about 7,000 cells undergoing adipogenesis. We then plotted the slope of *Rev-Erb*α reporter dynamics and the slope of PPARG abundance versus time to examine how the step increase in the PPARG synthesis rate compares to the clock dynamics (Fig. 4 *A*, *Left*). Markedly, the analysis showed that the synthesis rate of PPARG increases more strongly during the rising phase of the *Rev-Erb*α sensor. When oscillations in the circadian clock are abolished by knocking down the key clock protein BMAL1, PPARG synthesis still increases overall in response to the differentiation stimulus, but not in an oscillatory fashion (Fig. 4 *A*, *Right*).

E-boxes are typically needed to control the circadian expression of genes (22). Since PPARG does not have BMAL1/CLOCK-regulated E-boxes in its promoter, it is puzzling what causes PPARG synthesis rate to oscillate in a circadian fashion. PPARG is the master transcriptional regulator of adipogenesis and is at the center of multiple positive-feedback loops (Fig. 4*B*) (7, 23). We considered that the circadian expression of PPARG might be indirectly driven by one of the PPARG feedback partners, which might be a direct target of BMAL1/CLOCK. We focused on CEBPA since it is the main positive-feedback partner of PPARG and is essential for maintaining PPARG expression (24). Furthermore, CEBPA has two E-boxes on its promoter (25), and its messenger RNA (mRNA) level was shown to oscillate in a circadian pattern in fibroblasts (25).

We first knocked down CEBPA and assessed the effect on PPARG circadian oscillations during adipogenesis. Knockdown of CEBPA dramatically reduced the expression of PPARG (Fig. 4*C*), as well as the amplitude of the slope of the circadian oscillations (*SI Appendix*, Fig. S5), supporting that CEBPA could be driving PPARG circadian oscillations. To test whether CEBPA expression is circadian in OP9 cells, we measured mRNA and protein-level changes of CEBPA using RT-qPCR and immunofluorescence, respectively. OP9 cells were first treated with a 1-h pulse of dexamethasone, a synthetic glucocorticoid, which has been shown to synchronize the circadian clock (10). We chose a short pulse duration of dexamethasone to allow for synchronization of the clock without inducing differentiation. After 16 h, we collected or fixed cells every 4 h from different wells and carried out RT-qPCR assays and immunofluorescence analysis to track the changes of CEBPA mRNA and protein. We found that CEBPA mRNA is indeed expressed in a circadian manner, as well as two well-established circadian regulators PER2 and PER3 (Fig. 4*D*). Consistent with a circadian regulation, CEBPA protein abundance also followed a circadian pattern based on a JTK_Cycle rhythmicity test (26) and peaked shortly after the peak in *Rev-Erb*α reporter expression (Fig. 4 *E* and *F*).

To understand the relationship between circadian expression of CEBPA and circadian PPARG expression, we used CRISPR-mediated genome editing to tag endogenous CEBPA with citrine (YFP) in OP9 preadipocyte cells (Fig. 4*G*). By stably transfecting the *Rev-Erb*α–mScarlet reporter into these cells, we could monitor CEBPA activity and circadian rhythms simultaneously in the same cell (Fig. 4 *G* and *H* and Movie S2). We induced adipogenesis of the dual reporter citrine–CEBPA/*Rev-Erb*α–mScarlet cells and calculated how the slope of the citrine–CEBPA level changes over time relative to the circadian cycles. Indeed, by performing the same slope analysis, as done in Fig. 4*A* for PPARG, we confirmed that the CEBPA synthesis rate increases in a circadian manner (Fig. 4 *I*, *Left*). It should be noted that the levels of both PPARG and CEBPA increase strongly during adipogenesis with or without circadian rhythms (Fig. 4 *A* and *I*, *Left*). However, when Bmal1 is knocked down, the rates of PPARG and CEBPA synthesis no longer increase in an oscillatory circadian manner (Fig. 4 *A* and *I*, *Right*).

To more precisely quantify when the rate of CEBPA protein synthesis increases, we measured the change in the citrine–CEPBA signal during each circadian period (measured by the peak-to-peak distance in the *Rev-ERB*α–mScarlet signal, 0 to 2π in Fig. 2*B*) for the cells in Fig. 4*H* to obtain an average citrine–CEBPA slope during one circadian period. We found that the citrine–CEBPA slope took the shape of a sine wave peaking at ∼1.4π (Fig. 4*J*). In contrast, the circadian oscillations of PPARG peaked slightly later, at ∼1.8π. Because the average circadian period is about 26 h (Fig. 1*G*), the gap between the peaks of CEBPA and PPARG slopes correspond to an approximate 5-h delay (Fig. 4*J*).

PPARG has both fast and slow feedback partners (7). CEBPA and FABP4 are examples of fast and slow positive feedback partners of PPARG, with half-lives of ∼3 and 30 h, respectively. It should be noted that whereas expression of PPARG is necessary and sufficient for adipogenesis, PPARG feedback partners, such as CEBPA and FABP4, have no ability to promote adipogenesis in the absence of PPARG (24, 27). The fast half-life of CEBPA expression allows CEBPA levels to increase rapidly within a 12-h circadian gating window and also to decrease rapidly within that same time window if the cell does not reach the threshold to differentiate (Fig. 4*I*). However, FABP4, with such a long 30-h half-life, should not be able to increase and decrease in a 12-h on-off, circadian manner and thereby should be unable to regulate circadian PPARG expression. To test for this, we stably expressed the *Rev-Erb*α–mScarlet reporter in cells in which endogenous FABP4 was tagged with a fluorescent protein citrine (YFP) at its C-terminal (Fig. 4*K*) and induced the cells to differentiate (Fig. 4*L*). As expected, since FABP4 is a downstream transcriptional target of PPARG, the FABP4 levels increased dramatically, similar to PPARG levels (Fig. 1*C*). However, unlike PPARG and CEBPA (Fig. 4 *A* and *I*), the rate of FABP4 protein synthesis did not show a circadian pattern (Fig. 4 *L* and *M*). Together, these results support that clock-induced expression during each rising phase of the *Rev-Erb*α reporter of a fast-feedback partner, such as CEBPA, but not a slow-feedback partner, such as FABP4, can drive circadian PPARG expression.

### Computational Modeling Identifies Four Requirements for Circadian Gating of Differentiation Commitment and Generating Daily Bursts of Cell Differentiation.

We had observed circadian expression of PPARG and CEBPA in a bulk cell population (Fig. 4 *A*, *I*, and *J*). However, to observe circadian oscillations in single cells is challenging due to low signal-to-noise and also because very strong increases of CEBPA and PPARG during adipogenesis mask the much smaller amplitude of the circadian oscillations. Thus, to directly test whether expression of a fast-feedback partner such as CEBPA is what drives the daily bursts of differentiation commitments, we used computational modeling.

**Fig. 5.**
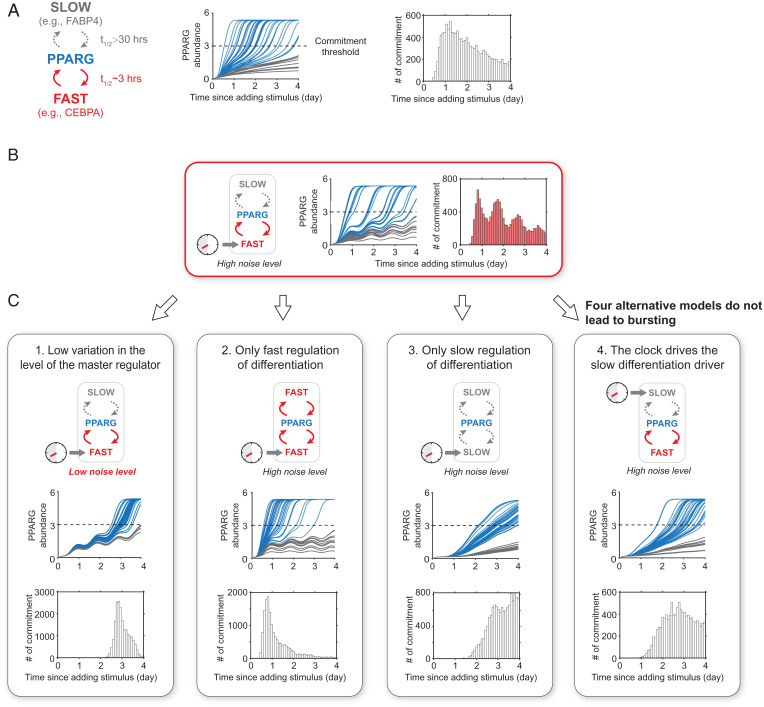
Four requirements for circadian gating of differentiation commitment and generating daily bursts of cell differentiation. (*A*) Quantitative simulations show that, due to the combined fast- and slow-feedback regulation, the abundance of PPARG increases slowly before and after a rapid switch that occurs as cells reach the threshold (dashed line). A total of 20,000 simulations were carried out, and the cell-to-cell variability was taken into account by randomly adding 30% log-normal noise to each simulation. Blue lines represent representative differentiated cells whose PPARG level passed the threshold line, with the gray lines representing undifferentiated cells. As consistent with the live-cell analysis, the time of differentiation commitment was defined as the time when the PPARG level reached the threshold. (*B*) Coupling the circadian clock to the fast regulator CEBPA in the model recapitulates the experimentally observed circadian bursts of cell differentiation. (*C*) Four simulations in which a different regulatory element was changed. High variation, slow and fast regulation of the master regulator, and coupling of the clock to the fast regulator are needed to generate daily bursts of differentiation commitments.

Our previous model to simulate PPARG dynamics in response to an adipogenic stimulus includes that differentiation commitment during adipogenesis is driven by fast and slow positive feedbacks centered on PPARG (7, 23) (Fig. 5 *A*, *Left*). We used the timescale of the CEBPA–PPARG feedback loop, *t*_1/2_ = 3 h, for the fast regulation and the timescale of the FABP4–PPARG feedback loop, *t*_1/2_ = 30 h, for the slow regulation (7). The simulations show that a typical PPARG time course follows an S-shaped curve, with the PPARG level in a cell first increasing relatively slowly after being induced to differentiate, but close to the threshold, there is a more rapid increase in PPARG level as cells irreversibly commit to differentiate (Fig. 5 *A*, *Center*). These simulations recapitulate the increases in PPARG observed in our experiments (Fig. 1 *C*, *E*, and *F*). Furthermore, the simulations recapitulate the known cell-to-cell variability (noise) in the fast- and slow-feedback circuits that regulate PPARG, which cause cells to reach the threshold stochastically at different times after adipogenesis is induced, in a manner that is evenly spaced over the several-day-long differentiation process (Fig. 5 *A*, *Right*, histogram) (7, 23, 28).

To now test for the effect of clock-driven regulation of a fast PPARG feedback partner, we next added a term to the model that superimposes oscillating circadian synthesis of a fast-feedback partner (Fig. 5*B*). Markedly, the model recapitulated the experimentally observed daily bursting behavior of cell differentiation (Fig. 2*D*). Thus, our simulations support that clock-driven expression of a fast PPARG feedback partner can drive circadian PPARG expression to generate daily bursts of cell differentiation over multiple clock cycles.

In the individual time courses of citrine–PPARG and *Rev-Erb*α–mScarlet dual reporter cells (Fig. 2*A*), we can see that there is variability when a cell irreversibly decides to commit to differentiate. In each clock cycle after differentiation is induced, only a subset of the progenitor cells reach the PPARG threshold to irreversibly commit. Because only some individual cells commit to differentiate in each of several consecutive circadian cycles, a bursting behavior can be observed at the population level. The reason only some cells reach the threshold at a given time is likely because of previously described cell-to-cell variability in the expression level of the master regulator PPARG when adipogenesis is induced (7, 23, 28). To validate the importance of cell-to-cell variation in PPARG expression for generating the bursting behavior, we reduced the variation of PPARG in the model. As shown in the traces and the single peak in the histogram in Fig. 5 *C*, *1*, reducing cell-to-cell variation of PPARG expression indeed leads to a loss of the bursting behavior.

In the model that generated bursting (Fig. 5*B*), we had included fast and slow regulation of the master differentiation regulator. We now wanted to understand if both speeds of regulation are needed to generate multiple daily bursts of cell differentiation. We thus modified the model to have only fast or only slow regulation of PPARG. As shown in the histogram in Fig. 5 *C*, *2*, with only fast regulators, almost all cells differentiated in the first clock cycle. As shown in the histogram in Fig. 5 *C*, *3*, with only slow regulators, cells committed to differentiate over a broader range of clock cycles. However, PPARG levels do not behave in a circadian manner since the slow regulation prevents rapid degradation of PPARG (7, 29), and, thus, PPARG levels cannot rapidly drop during the waking phase of the circadian clock. These results support that both slow and fast control of the master differentiation regulator are needed to generate circadian differentiation bursts over multiple days.

We also wanted to understand whether the circadian clock must drive the fast regulator of differentiation. As shown in Fig. 5 *C*, *4*, when we modified the model to have the clock drive the slow regulator, no circadian expression of PPARG was observed, and cells did not commit to differentiate in a circadian manner.

## Discussion

Our experiments and modeling showed that cell differentiation is gated, meaning that the circadian clock restricts differentiation commitment of individual preadipocytes almost exclusively to the rest phase each day. This circadian gating of individual cells leads to bursting behavior of cell-differentiation commitment at the level of the cell population, as seen in Figs. 2*D* and 5*B*.

Our results support that there are four requirements to generate circadian differentiation bursts over multiple days: 1) high cell-to-cell variability in expression of the master differentiation regulator; 2) a slow differentiation driver; 3) a fast differentiation driver; and 4) the circadian clock must be coupled to the fast differentiation driver. Cell-to-cell variability and slow regulation of PPARG prevent cells from all differentiating at one time or immediately after differentiation is induced. Fast regulation of PPARG allows differentiation commitment to occur rapidly—in ∼4 h (Fig. 1*F*)—which is well within the duration of the rest phase of the circadian clock. Coupling the clock to a fast differentiation driver like CEBPA allows PPARG levels in individual cells to be boosted rapidly toward the threshold during each rest phase of the clock. If an individual cell does not reach the threshold to differentiate within this gating window, the fast regulation of PPARG allows PPARG levels to quickly drop back down during the active phase of the clock, generating a circadian pattern of PPARG expression. Thus, coupling fast regulation of PPARG to the circadian clock explains the gating of differentiation commitment in individual cells to the rest phase of the circadian clock, and cell-to-cell variability and the slow regulation of PPARG explain why differentiation commitment is spread out over multiple clock cycles and can thus explain the bursting behavior at the population level.

Whereas we found that CEBPA likely mediates circadian gating of PPARG, results by us and others (23, 30, 31) suggest that CEBPA may not be the only fast regulator of circadian gating of differentiation commitment. For example, at the transcriptional level, Runx2, another feedback partner of PPARG (23), has been shown to be a target of the circadian clock and could potentially boost PPARG expression just like CEBPA (31). Moreover, other fast regulation of PPARG could be through posttranslational regulation, such as binding of Per2, which has been shown to directly regulate the activity of PPARG (32).

As was shown in Fig. 2*D*, not all cells will commit in the “right” phase due to cell-to-cell heterogeneity (23, 28). One advantage is that it facilitates adapting to a new schedule. For example, if one has to adapt to night-shift work or to another time zone because of jet lag, cells have the ability to commit to differentiate during the individual’s shifted circadian phases. On the other hand, a disadvantage of cells differentiating during the wrong phase is that these cells are then not able to coordinate with other circadian-regulated system, such as metabolism, DNA repair, and cell proliferation. Also, if too many cells commit to differentiate at the wrong phase, it would make adipogenesis uncontrollable.

Overall, our study provides direct evidence that the circadian clock restricts fat-cell differentiation commitment to the rest phase each day. Differentiation commitment involves major transcriptional and chromatin changes. Clock-mediated restriction of differentiation commitment to the rest phase, during which metabolic activity is likely lower than in the wake phase, may help to increase the reliability of cell differentiation. Our study also defines the differentiation-system criteria needed to generate the observed daily bursts of cell differentiation. Other cell-differentiation systems, such as Th17 and skeletal muscle differentiation, may employ similar circadian bursting and gating-regulation mechanisms (4, 33).

## Materials and Methods

### Generation of Citrine–PPARG/*Rev-Erbα*–mScarlet Dual-Readout Cell Lines.

To generate a stable live cell sensor for *Rev-Erbα* activity, the entire open reading frame of the Rev-VNP expression cassette described in a previous work (10) was PCR-amplified in addition to a 1-kb region upstream of the start codon containing the *Rev-Erbα* promoter elements. The amplified fragments were cloned by using Gibson assembly into a Piggyback expression backbone PB-CMV-MCS-EF1α-Puro vector (System Biosciences), which had been previously modified with PGK-Blasticidin in place of pEF1α-Puromycin and linearized by using SfiI/XbaI. The assembled construct, PB-REVERBA-Venus-NLS-PEST, was then digested with NotI and SalI to swap Venus fluorophore with a GBlock-Gene Fragment (IDT) containing mScarlet, which was inserted by using Gibson assembly to generate PB-REVERBA-mScarlet-NLS-PEST. The PB-REVERBA-mScarlet-NLS-PEST construct was then transfected into OP9 cells already expressing endogenously tagged citrine (YFP)–PPARG (7) using Lipofectamine 2000 (Thermofisher). Cells were selected for 48 h posttransfection using 10 μg/mL Blasticidin (Invivogen) for 10 d and fluorescence-activated cell sorting (FACS) sorted for mScarlet (RFP). To facilitate cell tracking in microscopy experiments, cells were subsequently infected with lentivirus (PLV-H2B-mTurquoise) to introduce a nuclear marker and further FACS-sorted on CFP. During this process, single clones were also isolated, expanded, and tested for their ability to maintain proper circadian rhythmicity and differentiate into adipocytes upon DMI stimulation.

### Generation Citrine–CEBPA/*Rev-Erbα*–mScarlet Dual-Readout Cell Lines.

To generate OP9 cells in which endogenous CEBPA is tagged at the N terminus with citrine, we followed the same protocol used to tag the N terminus of endogenous PPARG in OP9 cells with citrine (YFP) (7). The nuclear marker (PLV-H2B-mTurquoise) and circadian reporter (PB-REVERBA-mScarlet-NLS-PEST) were then stably integrated into the citrine–CEBPA cells. Single clones were isolated and tested in the same manner as described above.

### Generation FABP4–Citrine/*Rev-Erbα*–mScarlet Dual-Readout Cell Lines.

To generate OP9 cells in which endogenous FABP4 is tagged at the C terminus with citrine, we followed the same protocol used to tag the C terminus of endogenous PPARG in OP9 cells with citrine (YFP) (7). The nuclear marker (PLV-H2B-mTurquoise) and circadian reporter (PB-REVERBA-mScarlet-NLS-PEST) were then stably integrated into the FABP4–citrine cells.

### Cell Culture and Differentiation.

The wild-type OP9 cells and the dual-readout OP9 cells were maintained according to published protocols (7). Briefly, the cells were cultured in full growth medium consisting of minimal essential medium-α (MEM-α) (ThermoFisher Scientific) containing 1 unit/mL penicillin, 1 mg/mL streptomycin, and 292 μg/mL l-glutamate supplemented with 20% fetal bovine serum (FBS). To induce differentiation, 100 nM rosiglitazone (Cayman) or the adipogenic mixture (DMI), consisting of dexamethasone (1 μM; Sigma-Aldrich), IBMX (250 μM; Sigma-Aldrich), and insulin (1.75 nM; Sigma-Aldrich), was used. For live-imaging experiments, the differentiation stimuli were added to Fluorobrite Dulbecco’s modified Eagle medium (DMEM) (ThermoFisher Scientific), supplemented with 10% FBS, and then cells were continually imaged for 4 d. The small molecule LH846 (Cayman) was used at a concentration of 4 μM. For fixed-cell experiments, stimuli were added to MEM-α (ThermoFisher Scientific) supplemented with 10% FBS for 2 d and then removed and replaced with fresh medium containing 1.75 nM insulin (Sigma-Aldrich) and 10% FBS for another 2 d.

### Live-Cell Imaging.

A total of 15,000 cells per well were plated 24 h prior to imaging in full growth medium in Ibidi μ-Plates (catalog no. 89626). Before image acquisition, the full growth medium was aspirated and replaced with fresh Fluorobrite DMEM (ThermoFisher Scientific) supplemented with 10% FBS to reduce background fluorescence. Live-cell imaging was conducted by using the ECLIPSE Ti2 inverted microscope (Nikon) with a 10× Plan Apo 0.45-numerical aperture (NA) objective. Cells were imaged in a humidified 37 °C chamber at 5% CO_2_, and images were taken every 12 min in three fluorescent channels: CFP, YFP, and RFP. Total light exposure time was kept to less than 600 ms for each time point. Four nonoverlapping sites in each well were imaged.

### Immunofluorescence Staining and Imaging.

Cells were plated in Costar 96-well plates (catalog no. 3904) and fixed with 4% paraformaldehyde in phosphate-buffered saline (PBS) for 15 min at room temperature, followed by four washes with PBS using an automated plate washer (Biotek). Cells were then permeabilized with 0.1% Triton X-100 in PBS for 20 min at 4 °C, blocked with 5% bovine serum albumin (BSA; Sigma-Aldrich) in PBS for 1 h at room temperature, and stained with primary antibody (rabbit anti-CEBPA, 1:1,000, Santa Cruz Biotechnology catalog no. sc-61; rabbit anti-PPARG, 1:1,000, Cell Signaling catalog no. 2442; mouse anti-PPARG, 1:1,000, Santa Cruz Biotechnology catalog no. sc-7273; goat anti-FABP4, 1:1,000, R&D Systems catalog no. AF1443; mouse anti-Adiponectin, 1:1,000, Abcam catalog no. ab22554; and goat anti-Glut4, 1:1,000, Santa Cruz Biotechnology catalog no. sc-1608) in 1% BSA overnight at 4 °C. After four washes, cells were incubated with Hoechst (1:2,000) and secondary antibody (Alexa Fluor 647 anti-rabbit, 1:1,000) in the dark in 1% BSA for 1 h at room temperature. Prior to imaging, cells were washed four times with PBS. For assays involving EdU staining, cells were treated with 10 μM EdU for about 15 min prior to fixation. Fixed-cell imaging was conducted by using an ImageXpress MicroXL automated epifluorescence microscope (Molecular Devices) with a 10× Plan Apo 0.45-NA objective. Several nonoverlapping sites in each well were imaged.

### Image Processing and Analysis.

Fluorescence images were analyzed by using custom scripts and the MACKtrack package (8) in MATLAB R2021a (MathWorks). Cells were segmented and tracked for their nuclei based on either Hoechst staining (fixed-cell imaging) or H2B-Turquoise marker (live-cell imaging). Flat-field correction for each channel was carried out prior to signal measurement. Quantification of PPARG, CEBPA, mScarlet, FABP4, and EdU in cells was based on quantification of mean fluorescence signal over nuclei. Each single-cell trajectory of PPARG, CEBPA, FABP4, mScarlet, and EdU was smoothed by using a moving average filter with a 6-h span. The slope of PPARG, CEBPA, FABP4, and *Rev-Erbα*–mScarlet at each time point was calculated by using a linear fit to 8-h segments of the trajectory (±4 h). The total H2B-mTurquoise fluorescence was calculated by summing all pixel values of H2B-mTurquoise images over the nuclear region. A cell division was called when both of the two nearest future neighbors had the total nuclear H2B signals that were 45 to 55% of the original cell (18).

### Calculating the Threshold for Differentiation Commitment.

The terminal fate for a given cell was scored as differentiated or undifferentiated based on if its terminal PPARG expression level was above or below a preset cutoff value. The preset cutoff value (the ground truth) was set so that there will be less than 3% of control (dimethyl sulfoxide [DMSO]) cells scored as terminally differentiated cells (*SI Appendix*, Fig. S6). Then, to determine when cells commit to the differentiated state, we tested a series of thresholds to predict cells’ terminal fates before the experiment ended. For a given threshold value, cells would be predicted as differentiated if their nuclear citrine–PPARG time courses reached above the threshold value prior to the end of the experiment. The false-positive rate and true-positive rate of the predictions were calculated based on the ground truth. Next, we plotted the ROC for all the threshold values and selected the one as the optimal threshold whose point on the ROC was closest to the corner point (0, 1). This optimal threshold can maximize the difference between the true-positive rate and false-positive rate for predicting cell-fate choice. The time of differentiation commitment for each terminally differentiated cell was determined as the moment when its nuclear citrine–PPARG time course crossed the optimal threshold for the first time.

### siRNA-Mediated Gene Silencing.

siRNA targeting Bmal1 and the AllStars Negative Control siRNA were purchased from Qiagen. For siRNA knockdown in the live-cell imaging experiments, dual-readout cells were transfected by reverse-transfection using Lipofectamine RNAiMax (Invitrogen). Briefly, our reverse-transfection protocol per well was as follows: mix 40 μL of Opti-MEM medium (ThermoFisher Scientific), 0.4 μL of a 10 μM siRNA stock solution, and 1 μL of RNAiMax. The solution was incubated at room temperature for 10 min, and then 160 μL of culture medium containing the desired number of cells per well was added. Then, the entire (∼200 μL) volume was plated into 1 well of an Ibidi 96-well μ-plate. The siRNA/RNAiMax mixture was left on the cells for 24 h before being aspirated away and replaced with fresh Fluorobrite DMEM supplemented with 10% FBS prior to imaging.

### RT-qPCR.

RNA was extracted by using the RNeasy Mini Kit (QIAGEN). Complementary DNA synthesis was performed by using the qScript kit (Quantabio, catalog no. 101414-098), according to the manufacturer’s instructions. qPCR was performed by using the GoTaq qPCR Master Mix (Promega, catalog no. M3001) in the LightCycler 480-Roche System, according to the supplier’s manual. Measurements were normalized to the housekeeping control (GAPDH). Primers used in this study were as follows: CEBPA-F: 5′-CAAGAACAGCAACGAGTACCG-3′, CEBPA-R: 5′-GTCACTGGTCAACTCCAGCAC-3′, PER2-F: 5′-GAAAGCTGTCACCACCATAGAA-3′, PER2-R: 5′-AACTCGCACTTCCTTTTCAGG-3′, PER3-F: 5′-AACACGAAGACCGAAACAGAAT-3′, and PER3-R: 5′-CTCGGCTGGGAAATACTTTTTCA-3′.

### Statistics.

Statistical parameters are reported in the figures and figure legends. All statistical analysis was performed in MATLAB R2021a (MathWorks) or R.

### Mathematical Modeling.


$$\frac{d\left[\mathrm{mPPARG}\right]}{dt}=b_{\mathrm{mPPARG}}+0.1\times \frac{{\left(\left[\mathrm{CEBPA}\right]+\left[\mathrm{SlowFBP}\right]\right)}^{4}}{1^{4}+{\left(\left[\mathrm{CEBPA}\right]+\left[\mathrm{SlowFBP}\right]\right)}^{4}}-d_{\mathrm{mPPARG}}\times \left[\mathrm{mPPARG}\right],$$

$$\frac{d[\mathrm{PPARG}]}{dt}=k_{t}\times \left[\mathrm{mPPARG}\right]+k_{\mathrm{inact}}\times \left[\mathrm{PPAR}G^{*}\right]-k_{\mathrm{act}}\times \left(b_{\mathrm{act}}+\frac{\mathrm{stim}}{1.2+\mathrm{stim}}\right)\times \left[\mathrm{PPARG}\right]-d_{\mathrm{PPARG}}\times [\mathrm{PPARG}],$$

$$\frac{d[\mathrm{PPAR}G^{*}]}{dt}=k_{\mathrm{act}}\times \left(b_{\mathrm{act}}+\frac{\mathrm{stim}}{1.2+\mathrm{stim}}\right)\times \left[\mathrm{PPARG}\right]-k_{\mathrm{inact}}\times \left[\mathrm{PPAR}G^{*}\right]-d_{\mathrm{PPARG}}\times [\mathrm{PPAR}G^{*}],$$

$$\frac{d\left[\mathrm{mCEBPA}\right]}{dt}=\mathrm{circadian}(t)\times \varepsilon_{1}\times \left(b_{\mathrm{mCEBPA}}+0.05\times \frac{{\left(w\times \left[\mathrm{PPARG}\right]+\left[\mathrm{PPAR}G^{*}\right]\right)}^{4}}{2^{4}+{\left(w\times \left[\mathrm{PPARG}\right]+\left[\mathrm{PPAR}G^{*}\right]\right)}^{4}}\right)-d_{\mathrm{mCEBPA}}\times \left[\mathrm{mCEBPA}\right],$$

$$\frac{d[\mathrm{CEBPA}]}{dt}=k_{t}\times \left[\mathrm{mCEBPA}\right]-d_{\mathrm{CEBPA}}\times [\mathrm{CEBPA}],$$

$$\frac{d[\mathrm{mSlowFBP}]}{dt}=\varepsilon_{2}\times (b_{\mathrm{mSlowFBP}}+0.003\times \frac{{\left(w\times \left[\mathrm{PPARG}\right]+\left[\mathrm{PPAR}G^{*}\right]\right)}^{4}}{3^{4}+{\left(w\times \left[\mathrm{PPARG}\right]+\left[\mathrm{PPAR}G^{*}\right]\right)}^{4}})-d_{\mathrm{mSlowFBP}}\times \left[\mathrm{mSlowFBP}\right],$$

$$\frac{d[\mathrm{SlowFBP}]}{dt}=k_{t}\times \left[\mathrm{mSlowFBP}\right]-d_{\mathrm{SlowFBP}}\times [\mathrm{SlowFBP}],$$

$$b_{\mathrm{mPPARG}}=0.003,$$

$$b_{\mathrm{mCEBPA}}=0.0013,$$

$$b_{\mathrm{mSlowFBP}}=0.00006,$$

$$b_{\mathrm{act}}=0.0005,$$

w=0.1,

$$k_{t}=0.0062,$$

$$k_{\mathrm{act}}=0.018,$$

$$k_{\mathrm{inact}}=0.025,$$

$$d_{\mathrm{mPPARG}}=0.0144,$$

$$d_{\mathrm{PPARG}}=0.0083,$$

$$d_{\mathrm{mCEBPA}}=0.0089,$$

$$d_{\mathrm{CEBPA}}=0.0033,$$

$$d_{\mathrm{mSlowFBP}}=0.00034,$$

$$d_{\mathrm{SlowFBP}}=0.0032.$$

1. The model above depicts the circuits of circadian regulation of adipogenesis based on a published model (7). The diagram of the model is shown in *SI Appendix*, Fig. S7.
2. The rate of change of the concentrations of seven species are calculated: PPARG mRNA, inactivated PPARG protein, activated PPARG protein (PPARG*), CEBPA mRNA, CEBPA protein, slow-feedback partner mRNA (*mSlowFBP*), and slow-feedback partner protein (*SlowFBP*).
3. All the variables are initialized to be zero.
4. The parameters are in relative units.
5. *k_t_* represents translation rate.
6. The factor *w* represents the relative activity of the original PPARG and the agonist-activated PPARG.
7. Degradation rates are adopted from previous measurements (7).
8. To mimic the adipogenic stimulus, stim is set to be one at day 0.
9. A cell is scored as differentiated if the concentration of total PPARG protein ([*PPARG*] + [PPARG*]) is above a cutoff determined by the bimodal expression at the end of the simulation.
10. The term *circadian(t)*, in the equation describing CEBPA transcription rate, is set to be a time-dependent cosine function.
11. In the scenario of slow–slow architecture (Fig. 5*C*), the degradation rates of CEBPA mRNA and CEBPA protein were replaced with that of slow-feedback partner mRNA (*mSlowFBP*) and slow-feedback partner protein (*SlowFBP*).
12. Lognormal noise (with mean = 0, SD = 30% for high noise level, and SD = 3% for low noise level) was randomly added to simulations through three independent noise factors (ε_1_ and ε_2_) before the synthesis terms of CEBPA mRNA and slow-feedback partner mRNA (7, 23).

## Supplementary Material

Supplementary File

Supplementary File

Supplementary File

## Data Availability

All data and custom software codes presented in this paper have been deposited at zenodo.org (https://doi.org/10.5281/zenodo.6886051) (34) .
